# Supplementary material for: Robust prediction of synthetic gRNA activity and cryptic DNA repair by disentangling cellular CRISPR cleavage outcomes
Source: Nat Commun. 2025 May 21;16:4717. doi: 10.1038/s41467-025-59947-0 (PMC12095496; doi:10.1038/s41467-025-59947-0)
Supplement: Supplementary file 1 — Supplementary Information [file 41467_2025_59947_MOESM1_ESM.pdf]

Supplementary Information

Robust prediction of synthetic gRNA activity and cryptic DNA repair by disentangling cellular CRISPR cleavage outcomes

Riesenberg et al.

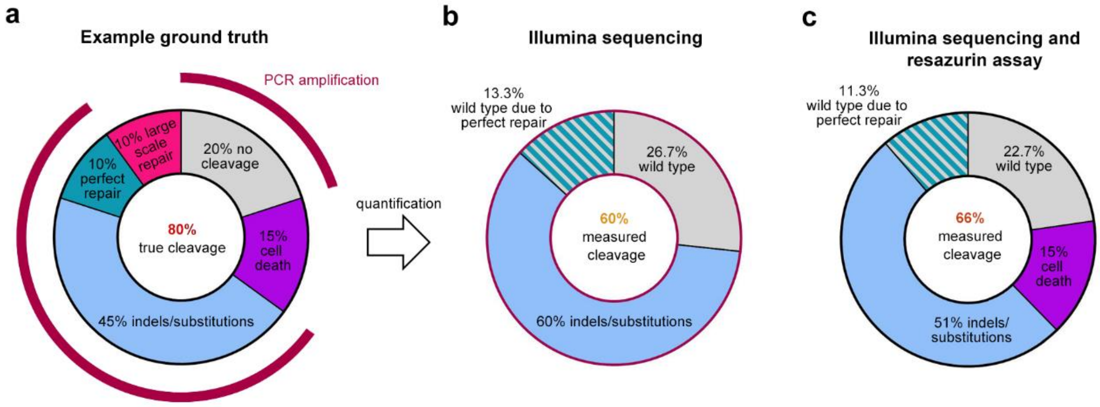

Supplementary Fig. 1: Cellular CRISPR cleavage outcomes and caveats of cleavage outcome detection. a) Example of percentage of cellular cleavage outcomes after CRISPR cleavage (20% uncleaved, 80% cleaved). Cleavage can result in cell death, indels/substitutions, perfect repair, or large-scale repair. **b**) Genome editing detected by next generation sequencing (NGS) corresponding to a. Only DNA from surviving cells can be sequenced, standard target PCR is unable to amplify large-scale repair events, and perfect repair results in sequencing reads that are identical to the uncleaved wild type sequence. This can overestimate indels while underestimating total cleavage. **c**) Combination of data from NGS and a resazurin assay, which quantifies cell death, can reduce the underestimation of CRISPR induced DNA cleavage.

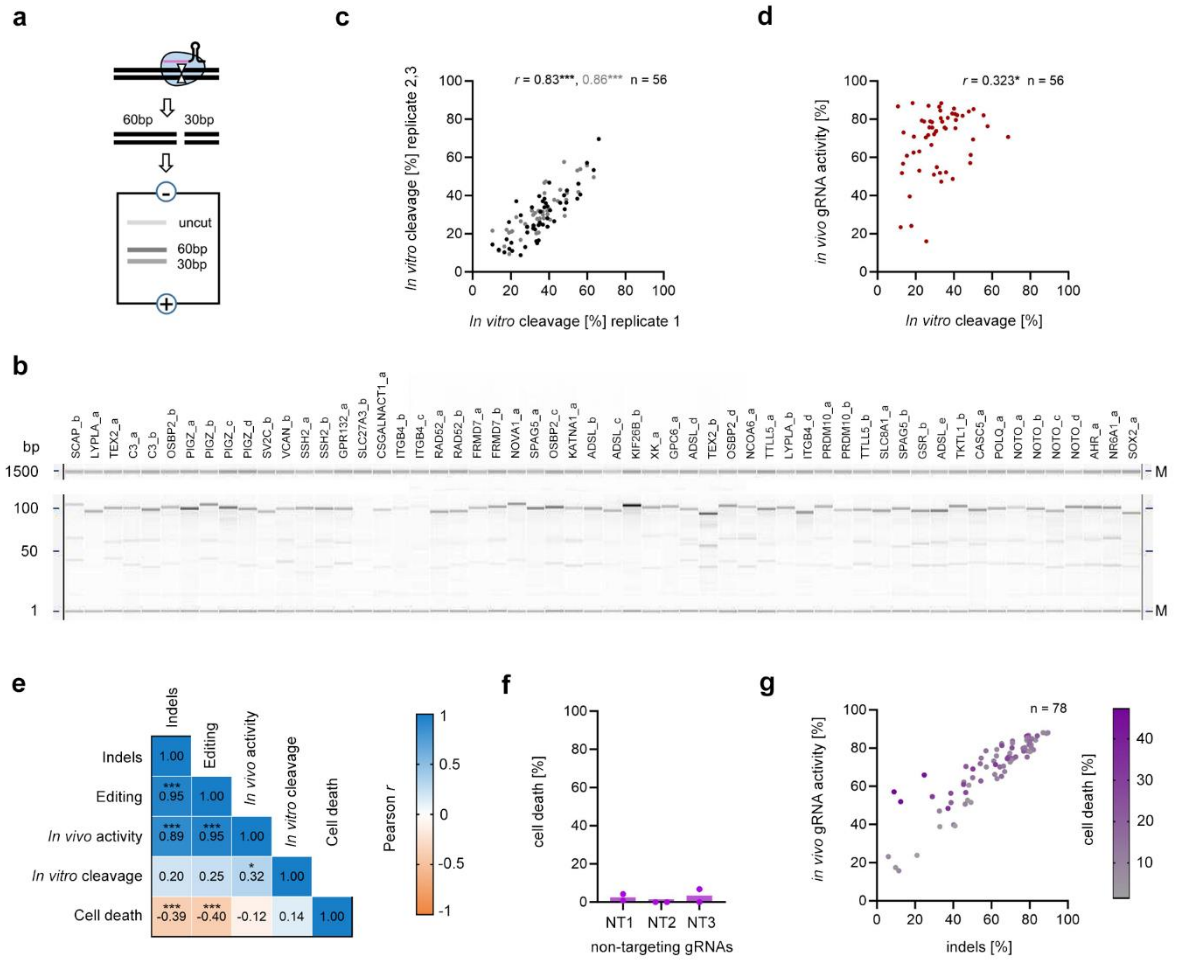

Supplementary Fig. 2: *In vitro* CRISPR-Cas9 cleavage screen. a) Schematic *of in vitro* cleavage detection. A successful CRISPR induced DSB results in two dsDNA molecules of 60 and 30bp, which are then separated by capillary electrophoresis. A DNA intercalating dye allows quantification of DNA band intensities of uncut DNA and cleavage products. Cleavage incubation was done for 16h at 37°C. **b**) Representative digital gel picture of capillary electrophoresis from *in vitro* CRISPR cleavage by different gRNAs. dsDNA sizes are stated on the left and upper and lower marker (1 and 1500bp) are indicated on the right. **c**) Scatter plots of replicates for percentage of *in vitro* cleavage. Pearson's r for correlation (two-tailed) of independent biological replicates (n = 3 for 47 targets, n =2 for 9 targets) is stated (***$p \leq 0.001$). **d**) Scatter plots of *in vitro* cleavage and *in vivo* gRNA activity. (*$p \leq 0.05$). Each dot represents the mean of independent biological replicates for one gRNA. **e**) Heatmap of Pearson's r for correlations between indels, editing, *in vivo* gRNA activity, *in vitro* cleavage, and cell death of the screened gRNAs (n = 78 *in vivo* screen, n = 56 *in vitro* screen) (*$p \leq 0.05$, ***$p \leq 0.001$). **f**) Cell death quantification for three non-targeting gRNAs. Independent biological replicates were performed (n = 2). **g**) Scatter plots of indels and *in vivo* gRNA activity, color coded by associated percentage of cell death (n = 78). Source data are provided as a Source Data file.

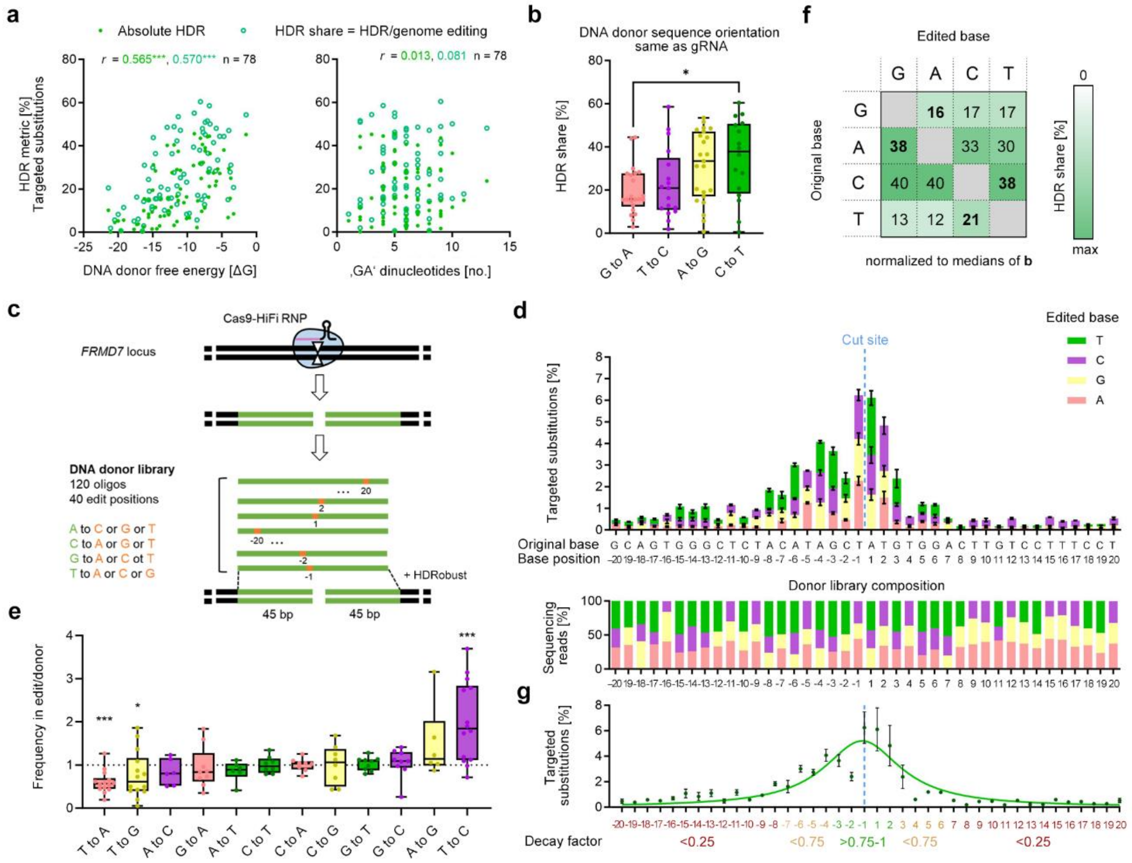

Supplementary Fig. 3: Features influencing HDR efficiency. a) Scatter plot of DNA donor (90nt ssDNA) free energy and percentage of targeted substitutions introduced by HDR (green dot), or HDR share defined as percentage of HDR of all editing events (open circles). Each symbol represents the mean of independent biological replicates (n = 3) for one gRNA (n = 78 targets). Pearson's r for correlation (two-tailed) is stated (***p ≤ 0.001). **b**) Percentage of HDR share for all transition mutations. Substitution events into adenine (rose), guanine (yellow), cytosine (purple), and thymine (green). Each dot indicates the mean of one target, boxes the 25th to 75th percentile, lines medians and whiskers extend from minimum to maximum values. DNA donors introduce a transition mutation directly at the cut site (position -1). Statistical significance using a one-way ANOVA with Tukey's multiple comparison test is stated (*p ≤ 0.05). **c**) Design of a DNA donor library editing approach that allows quantification of efficiency of all possible substitution mutations at different nucleotide distances to the *FRDM7* target cut site in 409B2 hiPSCs. The substance mix HDRobust [1] was used to increase overall HDR efficiency and prevent indel formation. **d**) The top panel shows percentages of targeted substitutions 20nt left and right of the cut site, respectively. The original base is stated on the x-axis. Substitution events into adenine (rose), guanine (yellow), cytosine (purple), and thymine (green) are shown. Independent biological replicates were performed (n = 3) and error bars show the s.e.m. The bottom panel shows the composition of the DNA donor library used for editing. **e**) Editing frequency of all possible substitutions normalized to their initial presence in the donor library. Each dot indicates the mean of for one position, boxes the 25th to 75th percentile, lines medians and whiskers extend from minimum to maximum values. The dotted line at 1 shows the expected frequency of substitutions that do not show a tendency to be enriched or depleted. Statistical significance using a one-way ANOVA with Tukey's multiple comparison test is stated (*p ≤ 0.05, ***p ≤ 0.001). **f**) Absolute HDR share in percentage for all possible substitutions from relative values from e by normalizing with absolute median values from b. **g**) Sum of targeted substitutions for each position. Shown is the mean of independent replicates (n = 3) and error bars show the s.e.m. The overall Lorentzian distribution (green line) is used to quantify the efficiency decay factor for each position, which is color coded below the x-axis. Source data are provided as a Source Data file.

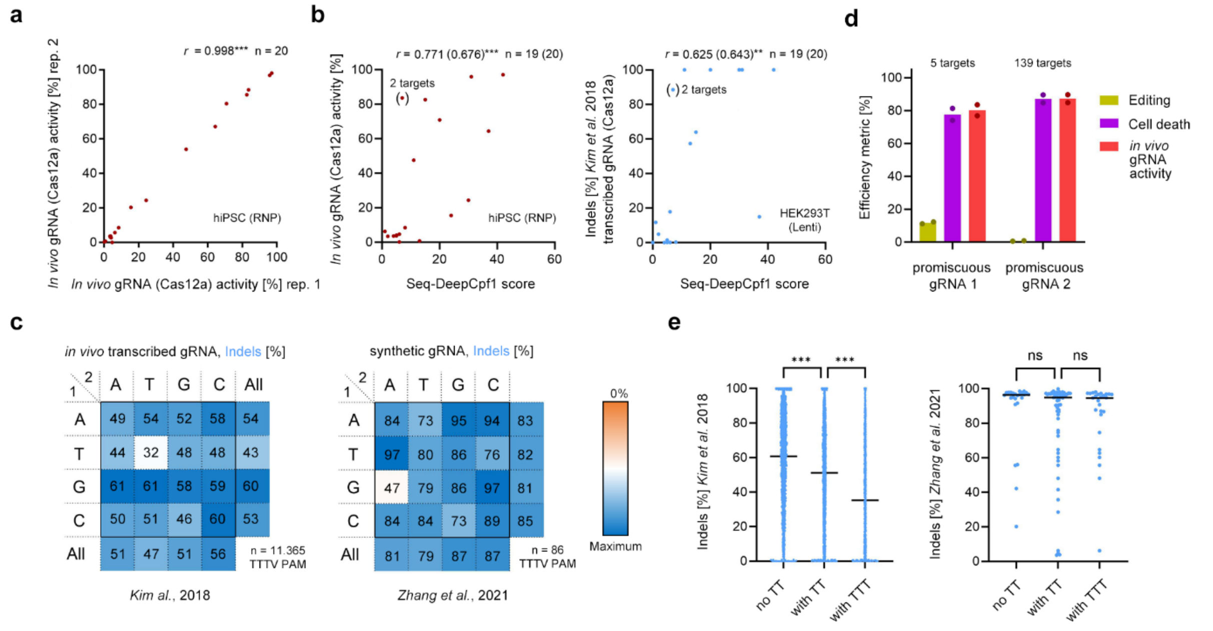

Supplementary Fig. 4: Cellular synthetic gRNA CRISPR-Cas12a (Cpf1) cleavage outcome screen and features influencing Cas12a gRNA activity. a) Scatter plots of replicates for *in vivo* gRNA activity (quantification of cell death and editing) in red, when editing with synthetic gRNA RNP (Cas12a-Ultra) and DNA donor in 409B2 hiPSCs. Pearson's r for correlation (two-tailed) of independent biological replicates (n = 2) is stated (n = 20 targets). Chosen targets have been previously used for editing and indel quantification in HEK293T cells [2]. **b**) Left panel: Scatter plot of the Seq-DeepCpf1 score [2] and measured *in vivo* Cas12a synthetic gRNA activity from a. Right panel: Scatter plot of the Seq-DeepCpf1 score and measured indels in HEK293T cells[2] when employing *in vivo* transcribed gRNAs. These 20 gRNAs are a subset of the 15,000 gRNAs used for training the Seq-DeepCpf1 prediction score. Pearson's r of the correlation is stated (**p ≤ 0.01, ***p ≤ 0.001). **c**) Heatmaps of mean indels induced by editing using Cas12a for all possible PAM-proximal dinucleotide combinations (positions 1 and 2 of the gRNA) of gRNAs with the preferred TTTV-PAM (V is any base but T). The left panel shows indels from a dataset of *in vivo* transcribed Cas12a gRNAs [2], and the right panel shows indels from a dataset of synthetic Cas12a gRNAs [3]. Both show limited influence of PAM-proximal nucleotides. **d**) Editing efficiency, cell death, and *in vivo* synthetic Cas12a gRNA activity for two targets that have identical off-targets in the human genome using RNPs in 409B2 hiPSCs. Independent biological replicates were performed (n = 2). **e**) Percentage of indels with gRNAs that have no 'TT' dinucleotide, at least one TT, or at least on TTT trinucleotide in the gRNA target sequence for datasets of *in vivo* transcribed Cas12a gRNAs (left panel), and synthetic Cas12a gRNAs (right panel), from c. Every dot indicates one gRNA and the horizontal line shows the respective means. Statistical significance using a one-way ANOVA with Tukey's multiple comparison test is stated (***p ≤ 0.001). Source data are provided as a Source Data file.

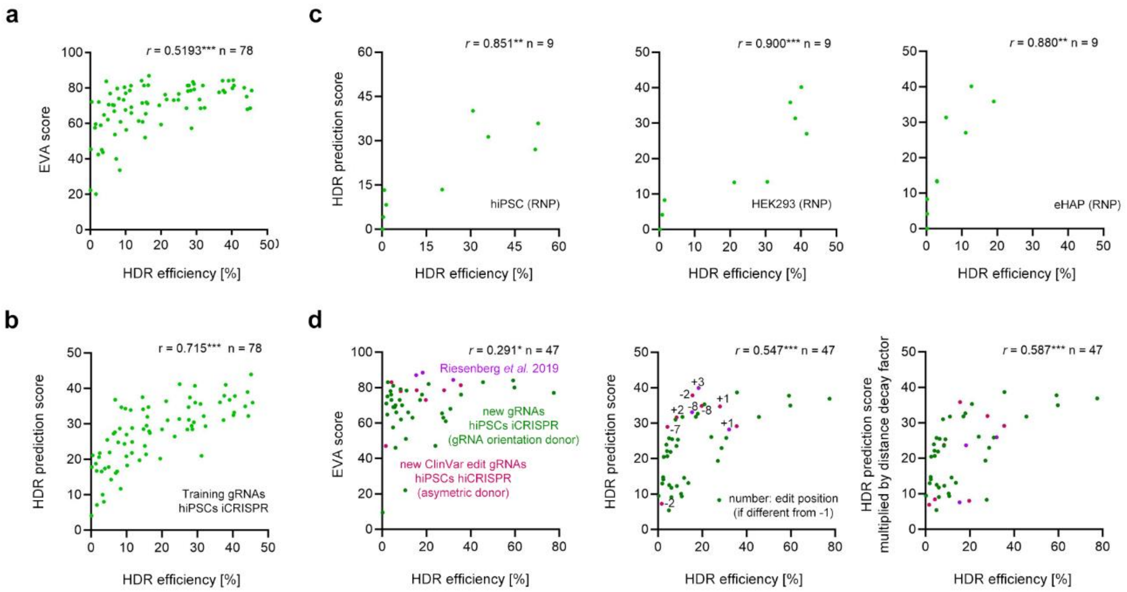

Supplementary Fig. 5: Prediction of HDR efficiency. a) Scatter plot of the EVA activity score and measured HDR efficiency from editing 409B2 iCRISPR hiPSCs. Each dot represents the mean of independent biological replicates (n = 3) for one gRNA (n = 78 targets). **b**) Scatter plot of the HDR prediction score and measured HDR efficiency of gRNA/donor pairs used for training. **c**) HDR prediction score and measured HDR efficiency for RNP based editing (n = 9) in 409B2 hiPSCs, HEK293, and eHAP. Each dot represents the mean of independent biological replicates (n = 2) for one gRNA. **d**) EVA score (left panel), HDR prediction score (middle panel), and HDR prediction score multiplied by distance efficiency decay (right panel) for gRNAs (n = 47) not used for training of the HDR prediction score in 409B2 iCRISPR hiPSCs. Green dots show donor designs identical to the initial screen (-1 position transition substitution, same DNA donor orientation as gRNA sequence), pinks dots show donor designs to install ClinVar substitutions (various positions and substitution types, asymmetric antisense DNA donor orientation), and purple dots show previously published HDR data in 409B2 hiPSCs [4]. An edit position with respect to the cut site different than -1 is stated in the middle panel. Each dot represents the mean of independent biological replicates (n = 3) for one gRNA. Pearson's r of the correlations (two-tailed) is stated (*p ≤ 0.05, **p ≤ 0.01, ***p ≤ 0.001). Source data are provided as a Source Data file.

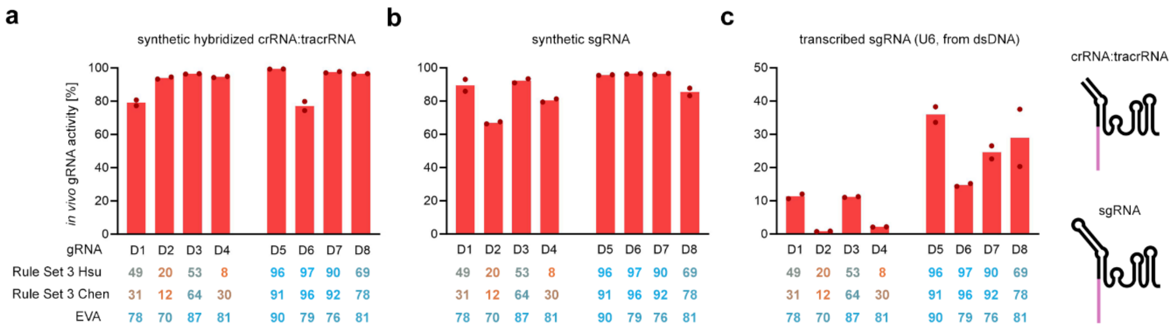

Supplementary Fig. 6: Comparison of gRNA architectures and delivery forms. *in vivo* gRNA activity of D1-D8 for editing in 409B2 hiPSCs using **a**) synthetic hybridized crRNA:tracrRNA, **b**) synthetic sgRNA, or **c**) sgRNA encoded in dsDNA and transcribed with a U6 promoter . Rule Set 3[5] percentile scores for both the Hsu and the Chen tracrRNA, as well as EVA scores are stated and color coded for predicted gRNA efficiency (orange: low, blue: high). sgRNAs contain an RNA loop that covalently connects the crRNA with the tracrRNA. For ease of comparison, the same data as in Fig.4c is shown in panel a. Independent biological replicates were performed (n = 2). Source data are provided as a Source Data file.

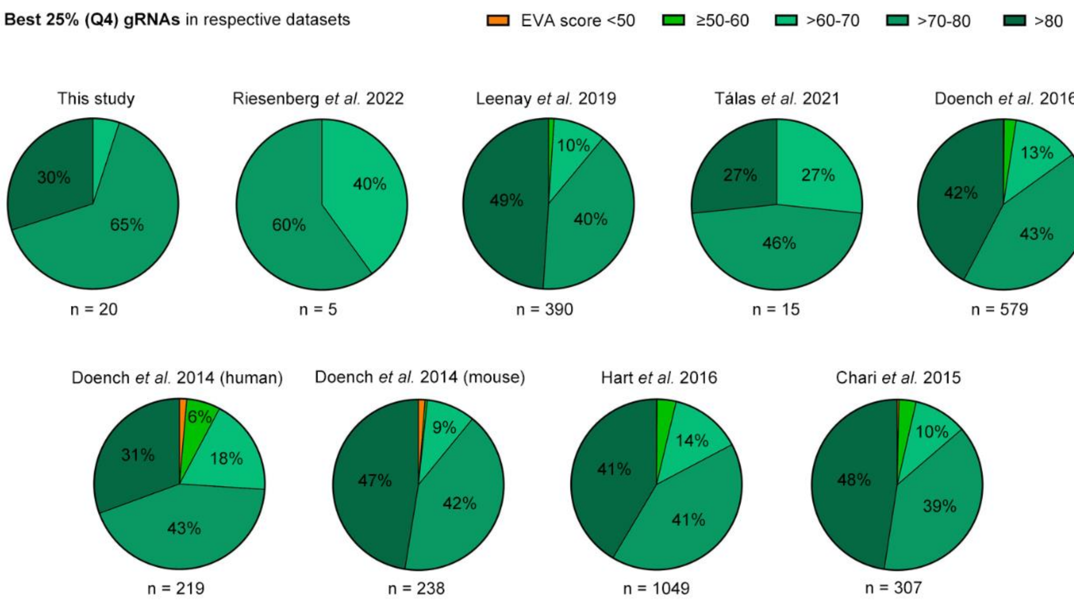

Supplementary Fig. 7: False positive bad gRNA scoring for published datasets. The best 25% (Q4) of gRNAs of published datasets and their portion of predicted to be efficient (green), or inefficient (orange), by the EVA score are shown. n numbers of gRNAs represented by the pie charts are stated. Source data are provided as a Source Data file.

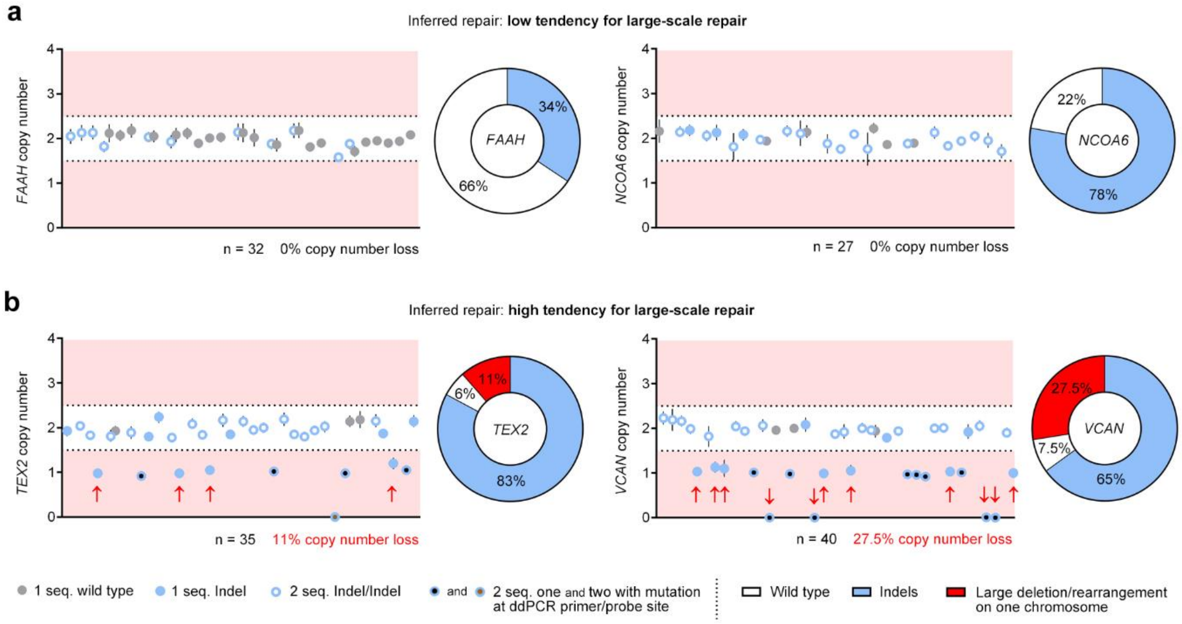

Supplementary Fig. 8: Validation of inferred large-scale repair tendency of gRNAs. Target site sequencing and droplet digital (dd) PCR copy number analysis of cellular clones after editing of with synthetic gRNA Cas9 RNP in 409B2 hiPSCs for two targets each inferred to have low tendency for large-scale repair (**a**), or high tendency for large-scale repair (**b**). For comparative screen inference see Fig. 5. The copy number of target sequences relative to the gene *FOXP2* in cellular clones is plotted as a filled or open circle when one predominant DNA sequence (seq.) (apparent homozygous) or two DNA sequences with a similar frequency (apparent heterozygous) were obtained, respectively. A black dot in a circle fill indicates an indel at the ddPCR primer/probe site that results in inability to amplify this locus for one chromosome. Cellular clones with copy number loss indicative of an on-target effect due to large-scale repair are labeled with red arrows. The measure of center for the error bars represents the ratio of the Poisson-corrected number of target to reference molecules multiplied by two for the diploid state of the reference gene. The error bars represent the 95% confidence interval of this measurement. The numbers of cellular clones analyzed and percentages of on-target effects are given. Pie charts give the percentage of genotypes of the cellular clones. Source data are provided as a Source Data file.

Supplementary References

1 Riesenberg, S. *et al.* Efficient high-precision homology-directed repair-dependent genome editing by HDRobust. *Nat Methods* **20**, 1388-1399 (2023). https://doi.org:10.1038/s41592-023-01949-1

2 Kim, H. K. *et al.* Deep learning improves prediction of CRISPR-Cpf1 guide RNA activity. *Nature Biotechnology* **36**, 239-+ (2018). https://doi.org:10.1038/nbt.4061

3 Zhang, L. *et al.* AsCas12a ultra nuclease facilitates the rapid generation of therapeutic cell medicines. *Nat Commun* **12**, 3908 (2021). https://doi.org:10.1038/s41467-021-24017-8

4 Riesenberg, S. *et al.* Simultaneous precise editing of multiple genes in human cells. *Nucleic Acids Res* **47**, e116 (2019). https://doi.org:10.1093/nar/gkz669

5 DeWeirdt, P. C. *et al.* Accounting for small variations in the tracrRNA sequence improves sgRNA activity predictions for CRISPR screening. *Nat Commun* **13**, 5255 (2022). https://doi.org:10.1038/s41467-022-33024-2
