# Supplementary material for: Robust prediction of synthetic gRNA activity and cryptic DNA repair by disentangling cellular CRISPR cleavage outcomes
Source: Nat Commun. 2025 May 21;16:4717. doi: 10.1038/s41467-025-59947-0 (PMC12095496; doi:10.1038/s41467-025-59947-0)
Supplement: Supplementary file 2 — Description of Additional Supplementary Files [file 41467_2025_59947_MOESM2_ESM.pdf]

Description of Additional Supplementary Files

Title: Supplementary Data 1

Description: gRNA activity screen data

Title: Supplementary Data 2

Description: gRNA activity model

Title: Supplementary Data 3

Description: HDR efficiency model

Title: Supplementary Data 4

Description: Collated published gRNA efficiency datasets

Title: Supplementary Data 5

Description: Comparative gRNA screen data with and without NHEJ inhibition

Title: Supplementary Data 6

Description: Oligonucleotides used in the study
